# Efficacy of Single Wound Infiltration With Bupivacaine and Adrenaline During Cesarean Delivery for Reduction of Postoperative Pain: A Randomized Clinical Trial

**DOI:** 10.1001/jamanetworkopen.2022.42203

**Published:** 2022-11-15

**Authors:** Gali Garmi, Mark Parasol, Noah Zafran, Michael Rudin, Shabtai Romano, Raed Salim

**Affiliations:** 1 Department of Obstetrics and Gynecology, Emek Medical Center, Afula, Israel; 2 The Ruth and Bruce Rappaport Faculty of Medicine Technion Haifa, Haifa, Israel; 3 Department of Anesthesia, Emek Medical Center, Afula, Israel

## Abstract

**Importance:**

Most women report moderate to severe pain after cesarean delivery. The extent of the ability of surgical wound infiltration with local anesthetic agents during cesarean delivery for the reduction of postoperative pain is uncertain.

**Objective:**

To examine the efficacy of single wound infiltration with bupivacaine and adrenaline during cesarean delivery for the reduction of postoperative pain.

**Design, Setting, and Participants:**

This randomized clinical trial was conducted between January 25, 2018, and May 30, 2020, at a university teaching hospital in Afula, Israel. A total of 288 women with singleton pregnancy who were scheduled for a planned cesarean delivery at term were randomized to receive single wound infiltration with bupivacaine and adrenaline during cesarean delivery (intervention group) or no single wound infiltration (control group) at a 1:1 ratio.

**Interventions:**

In the intervention group, the subcutaneous layer was infiltrated on both sides of the wound by a mixture of bupivacaine and adrenaline before wound closure. Other perioperative techniques were similar between the groups.

**Main Outcomes and Measures:**

The primary outcome was mean pain intensity over the course of 24 hours after the operation, as measured by a visual analog scale (VAS) score ranging from 0 to 10 (with higher scores indicating greater pain intensity). To detect a mean (SD) reduction of 1 (3) points in the VAS score in the intervention group, 286 women were needed in total. Secondary outcomes included VAS score greater than 4 (indicating moderate pain) at 2 hours after the operation, use of rescue opioids, maternal satisfaction with the pain management procedure (using a scale of 1-5, with higher scores indicating greater satisfaction), duration of the operation, scar complications (hematoma, infection, and separation), and length of stay.

**Results:**

Among 288 women (mean [SD] age, 32.5 [5.1] years; all of Arab or Jewish ethnicity), 143 were randomized to the intervention group, and 145 were randomized to the control group. Demographic and obstetric variables were similar between groups. The primary outcome (VAS pain score) was significantly lower in the intervention group (mean [SD], 2.21 [0.56]) compared with the control group (mean [SD], 2.41 [0.73]; *P* = .02). In the intervention group, 11 women (7.7%) had a VAS score greater than 4 at 2 hours compared with 22 women (15.2%) in the control group (odds ratio, 0.47; 95% CI, 0.22-1.00; *P* = .05). In addition, compared with the control group, the intervention group had significantly lower postpartum use of rescue opioid analgesics (19 women [13.3%] vs 37 women [25.5%]; *P* = .009) and greater satisfaction with pain management (mean [SD] score, 4.65 [0.68] vs 4.44 [0.76]; *P* = .007). In the intervention vs control groups, duration of the operation, scar complications (hematoma, infection, or separation), and length of stay were comparable.

**Conclusions and Relevance:**

In this study, wound infiltration with a single administration of bupivacaine and adrenaline during cesarean delivery reduced postoperative pain and opioid use and may have improved maternal satisfaction with pain management. These findings suggest the technique is efficacious, safe, and easy to perform.

**Trial Registration:**

ClinicalTrials.gov Identifier: NCT03395912

## Key Points

**Question:**

What is the efficacy of single wound infiltration with bupivacaine combined with adrenaline during cesarean delivery for the reduction of postoperative pain scores and opioid use?

**Findings:**

In this randomized clinical trial that included 288 women who underwent cesarean delivery at term, single wound infiltration with bupivacaine and adrenaline significantly reduced postoperative pain and postpartum use of rescue opioid analgesics. There were no intervention-related adverse effects.

**Meaning:**

These findings suggest intraoperative infiltration of the wound with single administration of bupivacaine and adrenaline in women undergoing a cesarean delivery is efficacious, safe, and easy to perform.

## Teaser

This randomized clinical trial assesses the efficacy of single wound infiltration with bupivacaine combined with adrenaline during cesarean delivery for the reduction of postoperative pain among women with singleton pregnancy who underwent cesarean delivery at term.

## Introduction

Cesarean delivery is one of the most frequently performed abdominal operations globally.^1^ Moderate to severe pain lasting at least 48 hours after cesarean delivery has been reported by most women.^2,3,4^ Achievement of adequate pain relief is important for adequate mother-child bonding, breastfeeding, early ambulation, timely hospital discharge, positive birth experience, and satisfaction.^5,6^

Treatment of pain related to cesarean delivery involves different strategies. Nonopioid systemic analgesic agents are widely used in different regimens. Nonetheless, opioid-sparing agents are occasionally not potent enough to achieve adequate pain control after cesarean delivery,^7^ and opioids are universally used. Consequently, several studies^8,9,10,11,12,13^ have reported that excessive amounts of opioids are being prescribed to women after cesarean delivery. These agents are associated with numerous adverse effects, and the risk of chronic use after cesarean delivery is of great concern.^8,9,10,11,12,13^

Infiltration of the wound with local anesthetics is one of the techniques that has been used in a range of surgical procedures for postoperative pain control. The technique affects somatic pain created by the surgical wound without producing major adverse effects.^14^ A meta-analysis of randomized clinical trials^8^ that investigated use of the technique during cesarean delivery suggested that wound infiltration with local anesthetics was associated with a reduction in postoperative opioid use. However, the benefit was noted only among women who did not receive intrathecal opioids, and pain scores did not consistently improve. Moreover, the studies included were small, compared different dosages, and had inconsistent results, and indirect comparisons were performed for subgroup analyses.^8^

In this randomized clinical trial, we aimed to examine (1) the efficacy of single wound infiltration with bupivacaine combined with adrenaline during cesarean delivery for the reduction of postoperative pain scores and (2) the impact of this technique on postoperative opioid use. The trial population included women scheduled for a planned cesarean delivery using spinal anesthesia with intrathecal bupivacaine and fentanyl only.

## Methods

This randomized clinical trial was conducted at a single university teaching hospital in Afula, Israel, between January 25, 2018, and May 30, 2020. Ethical approval for the trial protocol (Supplement 1) was obtained from the institutional review board of Emek Medical Center on January 14, 2018. All individual participants provided written informed consent. A local data monitoring committee implemented quality control of screening, handling of data, and verification of adherence to protocol. The study followed the Consolidated Standards of Reporting Trials (CONSORT) reporting guideline for randomized clinical trials.

Women with singleton pregnancy who were at term (≥37 weeks’ gestation) and scheduled for a planned Pfannenstiel incision cesarean delivery using spinal anesthesia were included. Exclusion criteria included known allergy to adrenaline or bupivacaine, liver or kidney disease, antenatal diagnosis of major fetal malformations, and antepartum fetal death. On January 29, 2018, an amendment was submitted to the institutional review board to include maternal cardiac disease, chronic hypertension, pregestational diabetes, and hyperthyroidism in the exclusion criteria.

Women were recruited 1 to 3 days before the scheduled cesarean delivery during the routine preoperative assessment. Women who provided consent were randomized to receive single wound infiltration with bupivacaine and adrenaline during cesarean delivery (intervention group) or no single wound infiltration (control group) at a 1:1 ratio. In the surgical suite, participants received spinal anesthesia with 2 mL of 0.50% bupivacaine and 25 μg of fentanyl. General anesthesia was administered for cases in which spinal anesthesia was ineffective. The infiltrated medications were prepared by the anesthesiologist. First, 1 mg of adrenaline was diluted in 100 mL of normal saline. Next, 15 mL of this solution was mixed with 15 mL of 0.50% bupivacaine hydrochloride to obtain a solution containing 30 mL of adrenaline (1:200 000) and 0.25% bupivacaine. After closing the fascia, the subcutaneous layer was infiltrated by the obstetrician in charge (one of whom was R.S.) using a sterile 23-gauge needle along the line of the wound in 3 to 4 separate portions, 15 mL in total on each side. The wound was then closed. Infiltration was not performed in the control group. Other intraoperative and postoperative management techniques were similar in both groups. The specific material used for wound closure was not part of the trial protocol. Metal staples, running subcuticular sutures, or glue was used according to the surgeons’ preferences and women’s requests. Three senior surgeons (one of whom was R.S.) with a similar level of experience performed all of the operations.

In the recovery ward, all women received the same analgesia protocol, which included 1 tablet of oxycodone with acetaminophen. Additional analgesia, if required, was added until discharge to the maternity ward in the following order: 5 mg of intravenous (IV) morphine sulfate, 10 mg of IV paracetamol (Perfalgan; Bristol-Myers Squibb), 500 mg of paracetamol in tablet form, 500 mg of dipyrone in tablet form, and 100 mg of tramadol in tablet form. In the maternity ward, the protocol for analgesia control was based on a previous clinical trial^15^ performed at our institution and published before the initiation of this clinical trial. Details are described in the original publication.^15^ The protocol was based on fixed time intervals of analgesia administration rather than on-demand administration. In brief, all women received 100 mg of IV tramadol hydrochloride, a 500-mg tablet of paracetamol, and a 100-mg tablet of diclofenac at admission. For the next 48 hours, all women received 2 tablets consisting of 325.0 mg of paracetamol and 35.5 mg of tramadol (Zaldiar; Aspen Pharmacare) every 6 hours. At 12 hours, 24 hours, and 48 hours after admission, all women also received a 100-mg tablet of diclofenac. In cases in which this protocol was insufficient and a woman needed additional medication for pain relief, a rescue tablet of oxycodone with acetaminophen was administered up to 4 times per day.

On admission to the maternity ward, pain measurements were recorded every 2 hours for the first 6 hours, then every 6 hours until 24 hours after admission, according to the ward protocol. Pain was assessed by a 10-cm visual analog scale (VAS) ranging from 0 to 10, with higher scores indicating greater pain intensity. The policy at our institution is to encourage mobility at 7 to 8 hours after the operation. The urinary bladder catheter is usually withdrawn at this time.

### Clinical Trial Outcomes

The primary outcome was mean pain intensity over the first 24 hours after the operation, as measured by VAS score. Secondary outcomes included VAS score at each time point (2 hours, 4 hours, 6 hours, 12 hours, 18 hours, and 24 hours) after the operation; VAS score greater than 4 (indicating moderate pain) and VAS score greater than 7 (indicating severe pain)^16^ at 2 hours after the operation; need for rescue opioids; duration of the operation; time from the end of the operation to mobility; breastfeeding and time from the end of the operation to breastfeeding among women who breastfed after cesarean delivery; surgical scar complications, including hematoma, infection, and separation; maternal satisfaction with pain management; and length of stay after the operation. As part of the study protocol, each participant was asked to grade her satisfaction regarding pain management using a verbal rating scale from 0 (not satisfied) to 5 (highly satisfied) at 24 hours after the operation.

### Randomization

All women provided informed consent and were enrolled in the clinical trial before entry into the operating room. Women were randomly assigned to the intervention group or the control group at a 1:1 ratio. A researcher (R.S.) who was not otherwise involved in enrolling women in the trial prepared the computer-generated randomization in blocks of 10. The randomization list was concealed in a closed study box. Eligible women were assigned the next available sequence in the randomization list. Investigators and enrolled participants were unaware of the upcoming group assignments until the moment of assignment. Women and surgeons were aware of the group assignments when they were in the surgical suite. Medical staff in the recovery department and the maternity unit were blinded to group assignment. In addition, the statistician was blinded to the identity code of either group.

### Sample Size

The primary outcome was mean VAS score measured over the first 24 hours after admission to the maternity ward. The SDs of post–cesarean delivery VAS scores reported in the literature have ranged from 0.1 to 2.7.^8^ We assumed a large amount of variation within the groups; therefore, we used an SD of 3.0. Based on this assumption and to demonstrate a difference of 1 in the mean VAS score between the groups, 286 women (143 in each group) were required with power of 80% and 2-sided α = .05.

### Statistical Analysis

Student *t* tests or Wilcoxon independent sample tests were used for nonnormally distributed continuous data, and χ^2^ tests or Fisher exact tests were used for categorical data. Data were considered to be approximately normal if the skewness and kurtosis were between −2 and 2. The mean VAS score over the first 24 hours was calculated using the raw score, and differences were analyzed using Wilcoxon independent sample tests. Because the hourly VAS scores were not normally distributed, a log transformation was used to normalize the measure for all analyses. Mixed-model repeated-measures analysis of variance was used to test (log transformed) VAS scores over the first 24 hours using a first-order autoregressive covariance structure for time. Analyses were based on intention to treat. Statistical analyses were performed using IBM SPSS Statistics software, version 28 (IBM Corp), for Windows (Microsoft Corporation) and MedCalc software, version 13.1 (MedCalc Software). Statistical significance was set to 2-tailed *P* < .05.

## Results

Between January 2018 and May 2020, 351 women were assessed for eligibility. Of those, 46 women (13.1%) declined participation, and 17 women (4.8%) did not meet the inclusion criteria. Overall, 288 women (mean [SD] age, 32.5 [5.1] years; all of Arab or Jewish ethnicity) were randomized to the 2 groups (143 to the intervention group and 145 to the control group) (Figure). Maternal demographic, obstetric, and perioperative variables are shown in Table 1. All characteristics were comparable between the intervention and control groups.

**Figure.**
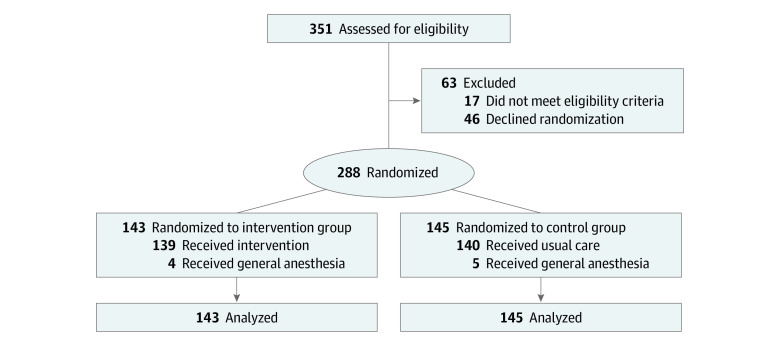
Study Flow Diagram

**Table 1.** Maternal Demographic, Obstetric, and Perioperative Characteristics

Characteristic	Participants, No. (%)	
	Intervention group (n = 143)	Control group (n = 145)
Age, mean (SD), y	32.6 (5.3)	32.4 (4.9)
Gravidity, mean (SD)	3.5 (1.8)	3.6 (1.9)
Parity, mean (SD)	2.0 (1.5)	1.9 (1.3)
Pregestational BMI, mean (SD)	26.2 (5.8)	26.3 (5.9)
Gestational age of neonate at birth, mean (SD), wk	38.7 (0.8)	38.7 (0.8)
Smoking	9 (6.3)	10 (6.9)
Background diseases		
Any disease	23 (16.1)	20 (13.8)
Thrombophilia	9 (6.3)	13 (9.0)
Thalassemia	2 (1.4)	1 (0.7)
Epilepsy	1 (0.7)	0
Hypothyroidism	7 (4.9)	5 (3.4)
Diabetes diagnosed in first trimester	4 (2.8)	0
Asthma	2 (1.4)	1 (0.7)
Other	2 (1.4)	2 (1.4)
Previous cesarean deliveries, mean (SD)	2.1 (1.2)	2.3 (1.4)
Gestational hypertension disorder	5 (3.5)	10 (6.9)
Diabetes during pregnancy	16 (11.2)	13 (9.0)
Indication for cesarean delivery		
Nonvertex presentation	18 (12.6)	23 (15.9)
Previous cesarean delivery	104 (72.7)	102 (70.3)
Previous anal sphincter injury	10 (7.0)	4 (2.8)
Macrosomia	5 (3.5)	6 (4.1)
Placenta previa	1 (0.7)	1 (0.7)
Previous myomectomy	1 (0.7)	0
Previous uterine perforation	0	1 (0.7)
Previous shoulder dystocia	2 (1.4)	2 (1.4)
Anesthesia		
Spinal	139 (97.2)	140 (96.6)
General (due to ineffective spinal)	4 (2.8)	5 (3.4)
Wound closure		
Metal staples	24 (16.8)	22 (15.2)
Subcuticular absorbable suture	28 (19.6)	19 (13.1)
Glue	91 (63.6)	104 (71.7)
Postpartum use of low–molecular-weight heparin	56 (39.2)	72 (49.7)

Abbreviation: BMI, body mass index (calculated as weight in kilograms divided by height in meters squared).

The VAS scores of the 2 groups are shown in Table 2. There was a significant decrease in mean VAS scores over the first 24 hours after admission in the intervention group (mean [SD] 2.21 [0.56]) compared with the control group (mean [SD], 2.41 [0.73]; mean difference, – 0.20; 95% CI, –0.35 to –0.05; *P* = .02). Mixed-model repeated-measures analysis of variance revealed a statistically significant difference in VAS scores during the first 24 hours after cesarean delivery between the 2 groups, with the intervention group reporting significantly lower VAS scores (estimated marginal mean, 2.11; 95% CI, 2.06-2.17) compared with the control group (estimated marginal mean, 2.24; 95% CI, 2.18-2.29; *P* = .007). In addition, there was a significant time effect, with a significantly lower VAS score in the intervention group at 24 hours (mean [SD], 1.99 [0.91]) than at 2 hours (mean [SD], 2.29 [1.30]; *P* = .05), 4 hours (mean [SD], 2.36 [1.35]; *P* = .04), and 6 hours (mean [SD], 2.37 [1.32]; *P* = .04) after admission. There was no significant interaction between group and time.

**Table 2.** Postoperative Visual Analog Scale Scores in the Maternity Unit^a^

VAS score	Mean (SD)		Mean difference (95% CI)
	Intervention group (n = 143)	Control group (n = 145)	
Within first 24 h	2.21 (0.56)	2.41 (0.73)	–0.20 (–0.35 to –0.05)
At 2 h	2.29 (1.30)	2.53 (1.40)	–0.24 (–0.56 to 0.07)
Score >4, No. (%)	11 (7.7)	22 (15.2)	0.47 (0.22 to 1.00)^b^
Score >7, No. (%)	1 (0.7)	1 (0.7)	1.01 (0.10 to 80.15)^b^
At 4 h	2.36 (1.35)	2.48 (1.55)	–0.11 (–0.45 to 0.22)
At 6 h	2.37 (1.32)	2.50 (1.47)	–0.13 (–0.45 to 0.20)
At 12 h	2.17 (1.13)	2.34 (1.42)	–0.18 (–0.47 to 0.12)
At 18 h	2.09 (0.97)	2.30 (1.24)	–0.21 (–0.47 to 0.04)
At 24 h	1.99 (0.91)	2.32 (1.50)	–0.32 (–0.61 to –0.04)

Abbreviation: VAS, visual analog scale.

^a^ VAS scores ranged from 0 to 10 on a 10-cm line, with higher scores indicating greater pain intensity.

^b^ Odds ratio.

The VAS scores were lower in the intervention group compared with the control group at 2 hours (mean difference, −0.24; 95% CI, −0.56 to 0.07), 4 hours (mean difference, −0.11; 95% CI, −0.45 to 0.22), 6 hours (mean difference, −0.13; 95% CI, −0.45 to 0.20), and 12 hours (mean difference, −0.18; 95% CI, −0.47 to 0.12), although the differences were not significant (Table 2). However, the mean VAS score at 24 hours after the operation was significantly lower in the intervention group (mean [SD], 1.99 [0.91]) compared with the control group (mean [SD], 2.32 [1.50]; mean difference, –0.32; 95% CI, –0.61 to –0.04). In the intervention group, 11 women (7.7%) had a VAS score greater than 4 at 2 hours after admission to the maternity ward compared with 22 women (15.2%) in the control group (*P* = .05; odds ratio [OR], 0.47; 95% CI, 0.22-1.00). The proportion of women who had a VAS score greater than 7 was comparable (1 woman [0.7%] in both groups; *P* = .99; OR, 1.01; 95% CI, 0.10-80.15).

The use of rescue analgesia in the recovery and maternity wards is shown in Table 3. The use of analgesia other than IV morphine in the recovery ward was significantly lower in the intervention group (135 women [94.4%]) compared with the control group (144 women [99.3%]; *P* = .02; OR, 0.12; 95% CI, 0.01-0.95). Furthermore, the proportion of women using rescue oxycodone with acetaminophen in the maternity ward was significantly lower in the intervention group compared with the control group (19 women [13.3%] vs 37 women [25.5%]; *P* = .009; OR, 0.45; 95% CI, 0.24-0.82).

**Table 3.** Use of Rescue Analgesia in the Recovery and Maternity Wards

Type of rescue analgesia	Participants, No. (%)		*P* value	OR (95% CI)
	Intervention group (n = 143)	Control group (n = 145)		
Recovery ward				
Intravenous morphine	83 (58.0)	89 (61.4)	.56	0.87 (0.54-1.43)
Any oral analgesia	135 (94.4)	144 (99.3)	.02	0.12 (0.01-0.95)
Maternity ward				
Oxycodone with acetaminophen	19 (13.3)	37 (25.5)	.009	0.45 (0.24-0.82)
No. of tablets, mean (SD)	0.14 (0.37)	0.26 (0.46)	.01	NA
Oxycodone dose, mean (SD), mg	0.7 (1.8)	1.3 (2.3)	.005	NA

Abbreviations: NA, not applicable; OR, odds ratio.

The mean (SD) duration of cesarean delivery was 42.8 (14.8) minutes in the intervention group vs 39.6 (11.5) minutes in the control group *(P =* .15) (Table 4). In the intervention and control groups, the proportion of women who breastfed postpartum and the time to breastfeeding initiation did not differ significantly. The time to mobility; incidences of scar hematoma, scar infection, and scar separation; and length of stay were also comparable. Women in the intervention group were significantly more satisfied with the pain management procedure during the 24 hours after admission compared with women in the control group (mean [SD] satisfaction score, 4.65 [0.68] vs 4.44 [0.76]; *P* = .007).

**Table 4.** Maternal Intraoperative and Postoperative Outcomes

Outcome	Participants, No. (%)		*P* value for intervention vs control group
	Intervention group (n = 143)	Control group (n = 145)	
Duration of operation, mean (SD), min	42.8 (14.8)	39.6 (11.5)	.15
Breastfeeding	123 (86.0)	131 (90.3)	.20
Time from cesarean delivery to first breastfeeding, mean (SD), h^a^	11.2 (10.7)	10.3 (10.7)	.54
Time from cesarean delivery to mobility, mean (SD), h	7.4 (5.1)	7.6 (7.4)	>.99
Satisfaction with pain management score, mean (SD)^b^	4.65 (0.68)	4.44 (0.76)	.007
Scar complications			
Hematoma	5 (3.5)	2 (1.4)	.25
Infection	3 (2.1)	0	.12
Separation	3 (2.1)	0	.12
Length of stay, mean (SD), d	3.4 (0.7)	3.6 (1.2)	.85

^a^ Time from cesarean delivery to first breastfeeding was recorded for 123 women in the intervention group and 131 women in the control group.

^b^ Satisfaction scores ranged from 1 to 5, with 1 indicating least satisfied and 5 indicating most satisfied.

## Discussion

In this randomized clinical trial, we examined the efficacy of single wound infiltration with bupivacaine combined with adrenaline before wound closure after cesarean delivery for the reduction of postoperative pain. The results showed that the technique reduced the mean VAS score during the first 24 hours after the operation. Although the difference between groups was small, the intervention also led to an almost 50% reduction (OR, 0.45) in the proportion of women with moderate pain at 2 hours after admission to the maternity ward despite lower use of rescue analgesia in the recovery ward. Furthermore, the use of rescue opioids was significantly reduced in the intervention group, and women in the intervention group were more satisfied with their postpartum pain management. Other intraoperative and postoperative outcomes that were examined were comparable between the groups. Mean surgical time was 3 minutes longer in the intervention group compared with the control group. However, this difference was not significant, and it was probably related to the additional time required for wound infiltration in the intervention group.

Postoperative pain after cesarean delivery is multifactorial, mainly related to a visceral component associated with uterine contractions and abdominal manipulation and to a somatic component associated with the wound. Techniques that apply local anesthetics affect the somatic component due to their ability to block pain impulses that originate from the surgical wound.^8^ Clinical studies in diverse surgical fields^17,18,19,20^ have suggested that the wound infiltration technique is effective, inexpensive, easy to learn, and safe and does not require special equipment. The technique has not led to increased rates of wound dehiscence or infection.^17,18,19,20^ Moreover, local anesthetics, typically bupivacaine, may impede antimicrobial activity. It has been reported that bupivacaine could inhibit the growth of numerous bacteria and fungi under various conditions.^21^

A Cochrane database review^22^ revealed that wound infiltration with local anesthesia was associated with decreased opioid consumption at 24 hours after cesarean delivery but did not reduce VAS scores. More recent meta-analyses from 2016^8^ and 2021^23^ concluded that wound infiltration with local anesthesia was associated with reductions in opioid consumption only among patients who did not receive intrathecal morphine. The number of studies included in those reviews, particularly studies assessing the use of wound infiltration in women receiving intrathecal opioids, was relatively small. There were differences among the studies in the anesthesia technique used, the type of local anesthetic agent used, the dose used, and the site of administration, resulting in significant heterogeneity in many of the outcomes examined.^8,23^ Compared with clinical trials included in previous meta-analyses and reviews,^8,23^ the present clinical trial is the largest, and the benefit of wound infiltration was confirmed.

In the present study, the mean VAS score was lower in the intervention group during the first 24 hours, and the effect was significant up to 24 hours after the operation. The duration of action and the half-life of bupivacaine hydrochloride when used for wound infiltration are approximately 12 hours and 3 hours, respectively.^19^ The duration of action when used for peripheral nerve blockade may be longer due to residual anesthetic effects through blockade of nerve fibers in the wound.^19,24^ Tverskoy et al^24^ reported that pain scores were reduced up to 48 hours after cesarean delivery. A reduction in postoperative pain for up to 10 days has also been reported.^25^ Moreover, a number of studies in other surgical fields^23,26,27,28^ have reported an additive benefit of combining adrenaline with local anesthesia for wound infiltration. Adrenaline, as applied in the present study, may have contributed to postoperative VAS score improvement, prolongation of the effect of bupivacaine, and further reduction in opioid use.^23,26,27,28^

Several analgesic methods, both pharmacological and nonpharmacological, may be used for pain management after cesarean delivery. Nonpharmacological methods, including transcutaneous electrical nerve stimulation^29^ and abdominal binder,^30^ are noninvasive and appear to be safe. Nevertheless, their efficacy is uncertain, and there is more evidence to support the efficacy of pharmacological methods. Several pharmacological techniques to reduce postsurgical pain scores and opioid use, including infiltration of local anesthetics to several sites other than the surgical wound with and without use of continuous infusions, have been reported. Two meta-analyses^8,31^ compared these techniques and found no significant advantages compared with single wound infiltration. A meta-analysis of randomized clinical trials^22^ compared wound infiltration with transversus abdominis plane block during cesarean delivery. The results suggested that both techniques were similar in terms of efficacy, safety, tolerability, and maternal satisfaction. Single administration represents the best-adapted technique owing to its safety, efficacy, and easy application.^8^ The use of a longer-acting injectable preparation of local anesthetic agents for wound infiltration, or adjustment of the dose to the patient's weight, are important areas for future research.

### Strengths and Limitations

This study has several strengths. The study is the largest randomized clinical trial of single wound infiltration among the cited studies, and there was high adherence to the study protocol during the trial. In addition, the trial was performed in a single institution, leading to strengths that include the same postoperative analgesia protocols, the same regional anesthesia protocol, the same surgical technique, and the same infiltration technique.

The study also has limitations. Although it is a randomized clinical trial, the study was not double-blind. Nevertheless, medical staff members in the recovery and maternity wards, where the main outcome was actually examined, were blinded to group assignment. Moreover, the statistician was blinded to the identity codes of the groups. In addition, the results may not be generalizable because this trial was conducted at a single center. Nevertheless, comparable protocols and outcomes have been reported in other institutions and for various surgical procedures. Furthermore, the results may not be generalizable to settings in which intrathecal morphine is used instead of fentanyl.

## Conclusions

This randomized clinical trial found that intraoperative infiltration of the surgical wound with single administration of local anesthetics during cesarean delivery provided additional analgesia and decreased opioid intake. Due to its efficacy and safety, this technique may be considered as standard analgesic practice among women undergoing a cesarean delivery. In addition, the technique is relatively easy to perform, and the mother-child relationship is not disrupted. Patients who do not receive intrathecal opioids or have general anesthesia for any reason may particularly benefit from this technique.

## Notes

Supplement 1.
Trial Protocol

Supplement 2.
Data Sharing Statement
