# Supplementary material for: Efficacy of Single Wound Infiltration With Bupivacaine and Adrenaline During Cesarean Delivery for Reduction of Postoperative Pain: A Randomized Clinical Trial
Source: JAMA Netw Open. 2022 Nov 15;5(11):e2242203. doi: 10.1001/jamanetworkopen.2022.42203 (PMC9667325; doi:10.1001/jamanetworkopen.2022.42203)
Supplement: Supplement 1. — Trial Protocol [file jamanetwopen-e2242203-s001.pdf]

Wound infiltration with bupivacaine and adrenaline during cesarean delivery to reduce postoperative pain: a randomized controlled trial

Protocol number: 0151-17-EMC.

Date of submission to IRB: October 26, 2017

Date of IRB approval of the final protocol: December 25, 2017

Date of approval of the EMC chair (final approval): January 14, 2018

Hypothesis

Subcutaneous infiltration with bupivacaine and adrenaline before wound closure during cesarean section (CS) will reduce the intensity of postoperative pain.

Objective

To examine the effect of single-shot wound infiltration with bupivacaine and adrenaline during CS on postoperative pain according to the visual analog scale (VAS).

Design

Randomized controlled trial

Study population

Term pregnant women scheduled for a planned CS under regional anesthesia.

Inclusion Criteria

- Planned CD
- Pfannenstiel wound CD
- Maternal age 18-60 years.
- Planned spinal anesthesia

Criteria for exclusion

- Allergy to adrenaline or bupivacaine
- Maternal diseases: cardiac, liver and kidney diseases, hyperthyroidism, chronic hypertension and pre-gestational diabetes.
- Multiple gestation
- Antenatal diagnosis of major fetal malformations
- Antepartum fetal death.
- Emergency CS.

Research plan

Women will be recruited prior to the scheduled CS during the routine preoperative assessment.

Consented women will be randomly assigned to the study (intervention) group or the control group.

In the study group, the wound will be infiltrated in both sides with 30 ml of 0.25% bupivacaine hydrochloride mixed with adrenaline 1:200000 before wound closure. Infiltration will not be performed in the control group. Other intra- and postoperative management will be similar in both groups.

On admission to the maternity ward, pain measurements will be recorded every 2 h for the first 6 h and then every 6 h until 24 h after admission, according to the ward protocol. Pain will be assessed by a VAS from 0 to 10, where a higher score indicates more pain.

At 24 hours after surgery the women will be asked to grade their satisfaction regarding pain management after surgery on a scale of 1 to 5 where 1 = not satisfied and 5 = very satisfied.

Demographic and obstetric data will be collected from the computerized sheets: woman's age, weight and height, smoking status, pre-pregnancy weight, pregnancy and birth number, number of previous caesarean sections, gestational age, background diseases, obstetric complications, use of anticoagulants, reasons for surgery, duration of surgery, type of anesthesia in surgery, and administration of analgesia at the recovery and maternity wards.

Primary outcome

The primary outcome will be the mean VAS scores after admission to the maternity ward.

Secondary outcomes will include VAS score at each time point, need for rescue opioids, duration of surgery, time from end of surgery to mobility, time from end of surgery to breastfeeding, hematoma or infection in the surgical scar, maternal satisfaction, and length of stay after surgery.

Research period

Women will be recruited prior to the scheduled CD during the routine preoperative assessment and will participate in the study until they discharge home after the surgery.

Randomization

Women will be randomly assigned to the study group or the control group at a 1:1 ratio. A computer-generated random allocation in blocks of ten will be created. The randomization list will be concealed in a closed study box. Eligible women will be assigned the next available sequence in the randomization list. Investigators and participants enrolled will be unaware of the upcoming group assignments until the moment of assignment.

Sample size

The standard deviation reported in the literature regarding post-cesarean VAS ranges from 0.1 to 2.7.* We assume a large amount of variation will exist within the groups, and therefore a standard deviation of 3.0 will be chosen. Based on this assumption and to demonstrate a difference of 1 in mean VAS score between the groups, 286 women (143 in each group) will be required with a power of 80% and a two-sided alpha of 5%. We are seeking approval to recruit up to 300 women in cases of a dropout.

* Adesope O, Ituk U, Habib AS. Local anaesthetic wound infiltration for postcaesarean section analgesia. A systematic review and meta-analysis. Eur. J. Anaesthesiol. 2016;33(10):731–742.

Statistics

T-test will be used to examine differences in continuous variables between the groups. For the purpose of comparing the categorical research variables, Chi-square or the Fisher exact test for small samples we be used. Other statistical testes will be used as needed.

Maintaining confidentiality

Patients' information will be stored separately in a sealed brown envelope and will not be included on the data collection sheet.

Amendments related to trial protocol:

Date: 29.01.2018

Addition to the exclusion criteria:

- Maternal cardiac disease
- Chronic Hypertension
- Hyperthyroidism
- Pre-gestational diabetes
