# Supplementary material for: Efficacy of Single Wound Infiltration With Bupivacaine and Adrenaline During Cesarean Delivery for Reduction of Postoperative Pain: A Randomized Clinical Trial
Source: JAMA Netw Open. 2022 Nov 15;5(11):e2242203. doi: 10.1001/jamanetworkopen.2022.42203 (PMC9667325; doi:10.1001/jamanetworkopen.2022.42203)
Supplement: Supplement 2. — Data Sharing Statement [file jamanetwopen-e2242203-s002.pdf]

# Data Sharing Statement

Garmi. Efficacy of Single Wound Infiltration With Bupivacaine and Adrenaline During Cesarean Delivery for Reduction of Postoperative Pain. *JAMA Netw Open*. Published November 15, 2022. doi:10.1001/jamanetworkopen.2022.42203

**Data**
**Data available:** Yes
**Data types:** Deidentified participant data
**How to access data:** salim_ra@clalit.org.il (Corresponding author)
**When available:** With publication

**Supporting Documents**
**Document types:** None

**Additional Information**
**Who can access the data:** Data collected for the study including individual participant data will be made available after publication by email from the corresponding author for researchers who provide a methodologically sound proposal for performing individual participant data meta-analysis.
**Types of analyses:** individual participant data meta-analysis.
**Mechanisms of data availability:** after approval of a proposal
**Any additional restrictions:** none
